# The association of angiotensin-converting enzyme with biomarkers for Alzheimer’s disease

**DOI:** 10.1186/alzrt257

**Published:** 2014-05-15

**Authors:** Hadassa M Jochemsen, Charlotte E Teunissen, Emma L Ashby, Wiesje M van der Flier, Ruth E Jones, Mirjam I Geerlings, Philip Scheltens, Patrick G Kehoe, Majon Muller

**Affiliations:** 1 Alzheimer Center & Department of Neurology, Neuroscience Campus Amsterdam, VU University Medical center, ZKH 4A.35, PO Box 7057, 1007 MB Amsterdam, the Netherlands; 2 Julius Center for Health Sciences and Primary Care, University Medical Center Utrecht, Utrecht, the Netherlands; 3 Department of Clinical Chemistry, Neurological Laboratory, VU University Medical Center, Amsterdam, the Netherlands; 4 Dementia Research Group, Institute of Clinical Neurosciences, School of Clinical Sciences, University of Bristol, Frenchay Hospital, Bristol BS16 1LE, UK; 5 Department of Gerontology and Geriatric Medicine, Leiden University Medical Center, Leiden, The Netherlands

## Abstract

**Introduction:**

Lower angiotensin-converting enzyme (ACE) activity could increase the risk of Alzheimer’s disease (AD) as ACE functions to degrade amyloid-β (Aβ). Therefore, we investigated whether ACE protein and activity levels in cerebrospinal fluid (CSF) and serum were associated with CSF Aβ, total tau (tau) and tau phosphorylated at threonine 181 (ptau).

**Methods:**

We included 118 subjects from our memory clinic-based Amsterdam Dementia Cohort (mean age 66 ± 8 years) with subjective memory complaints (n = 40) or AD (n = 78), who did not use antihypertensive drugs. We measured ACE protein levels (ng/ml) and activity (RFU) in CSF and serum, and amyloid β_1–42_, tau and ptau (pg/ml) in CSF.

**Results:**

Cross-sectional regression analyses showed that ACE protein level and activity in CSF and serum were lower in patients with AD compared to controls. Lower CSF ACE protein level, and to a lesser extent serum ACE protein level and CSF ACE activity, were associated with lower CSF Aβ, indicating more brain Aβ pathology; adjusted regression coefficients (B) (95% CI) per SD increase were 0.09 (0.04; 0.15), 0.06 (0.00; 0.12) and 0.05 (0.00; 0.11), respectively. Further, lower CSF ACE protein level was associated with lower CSF tau and ptau levels; adjusted B’s (95% CI) per SD increase were 0.15 (0.06; 0.25) and 0.17 (0.10; 0.25), respectively.

**Conclusions:**

These results strengthen the hypothesis that ACE degrades Aβ. This could suggest that lowering ACE levels by for example ACE-inhibitors might have adverse consequences for patients with, or at risk for AD.

## Introduction

Alzheimer’s disease (AD) is characterized by the accumulation of extracellular amyloid-beta (Aβ) plaques and intracellular neurofibrillary tangles (tau pathology), which are reflected by lower Aβ, and higher total tau (tau) and tau phosphorylated at threonine 181 (ptau) in cerebrospinal fluid (CSF) [1].

In the last decade, the role of the renin–angiotensin system (RAS) in the etiology of AD has received increasing attention. Inheritance of the I-allele – associated with lower plasma angiotensin-converting enzyme (ACE) levels [2] – was related to increased risk of AD [3,4], although these findings have not been supported by recent genome-wide association studies [5,6] and large haplotype studies [7]. Further, *in vitro* studies showed that ACE functions to degrade Aβ, and administration of ACE inhibitors promoted the accumulation of Aβ [8-10], while *in vivo* studies on various mouse models of AD showed indirect evidence that ACE can degrade Aβ (reviewed in [11]). Together, these data suggest that ACE is important for Aβ degradation, and hence low ACE activity can lead to increased Aβ-mediated neuronal damage, plaque accumulation, and risk of AD [12].

Few studies have examined the association between ACE and AD biomarkers in CSF [13,14]. Results on the association between the I-allele, or haplotypes, of the *ACE* gene, which have been associated with lower ACE levels, and CSF Aβ were inconclusive [13,14]. Further, one small-scale study found that administration of an ACE inhibitor did not influence AD biomarkers in CSF [15].

The current literature almost entirely comes from laboratory-based and genetic association studies. However, the laboratory-based studies are not comparable with the human disease state and the findings from genetic-association studies may be explained by linkage disequilibrium with the true risk factor [3]. Examining direct measures of ACE (such as serum or CSF ACE) in relation to CSF AD biomarkers might therefore be more clinically meaningful – especially since the widely prescribed antihypertensive ACE-inhibitors strongly inhibit ACE activity [16], thereby possibly not only reducing the unfavorable angiotensin (ANG)I to ANGII conversion, but also the favorable Aβ degradation.

The aim of the current study was to investigate the association of serum and CSF ACE protein levels and activity with CSF AD biomarkers – Aβ, tau and ptau – in a memory clinic cohort. It has increasingly been recognized that nearly all organs of the body have their own local paracrine-like RAS, with organ-specific functions [17]. The brain also has its own RAS system, acting largely independent of the peripheral RAS [17]. We therefore hypothesize that CSF and serum ACE reflect the activity in the brain RAS and peripheral RAS, respectively, and that CSF ACE activity and, to a lesser extent, serum ACE activity are associated with lower CSF Aβ, indicating worse Aβ-related pathology in the brain.

## Methods

### Study population

Patients were included from the memory clinic-based Amsterdam Dementia Cohort of the Alzheimer Center of the VU University Medical Center (VUMC). They underwent standard dementia screening including physical and neurological examination, as well as laboratory tests, electroencephalography, brain magnetic resonance imaging and comprehensive neuropsychological testing. The diagnosis of probable AD was made according to the National Institute of Neurological Disorders and Stroke–Alzheimer’s Disease and Related Disorders Association criteria [18] by consensus of a multidisciplinary team, without knowledge of CSF results and the apolipoprotein E (*APOE*) genotype. When the results of all examinations were normal, patients were considered to have subjective complaints (that is, criteria for mild cognitive impairment not fulfilled). Diabetes mellitus and hypercholesterolemia were defined based on self-reported medical history and medication use. Blood pressure was measured manually in a standardized manner using a sphygmomanometer with the patient in a sitting position after 5 minutes of rest. The level of education was classified using the seven-point rating scale of Verhage ranging from 1 (low, elementary school not completed) to 7 (high, university).

For the current study, patients using antihypertensive drugs were excluded, because some of these drugs strongly influence ACE levels [19]. Of the patients aged 50 to 80 years old with available CSF, we included 40 persons with subjective complaints (control group) and 78 persons with AD (AD group). The ethical review board of the VUMC approved the study and all subjects gave written informed consent.

### Sampling of blood and cerebrospinal fluid

A blood sample was taken, and after half an hour of clotting the samples were spun at 1,800 × *g* for 10 minutes at 4°C, then aliquoted into Sarstedt polypropylene cryovials and immediately stored at -80°C until further analyses. CSF was obtained by lumbar puncture between the L3/L4 or the L4/L5 intervertebral space, using a 25-gauge needle, and collected in 10 ml polypropylene tubes. Within two hours, 2 ml of CSF samples were centrifuged at 1,800 × *g* for 10 minutes at 4°C and stored at -20°C for analysis of CSF biomarkers within 2 months (see below). The remainder of the CSF was aliquoted in polypropylene tubes of 0.5 or 1 ml, and stored at -80°C until further analysis [20].

### *APOE* and Alzheimer’s disease cerebrospinal fluid biomarkers

DNA was isolated from 10 ml ethylenediamine tetraacetic acid blood and the *APOE* genotype was determined using the Light Cycler *APOE* mutation detection method (Roche Diagnostics GmbH, Mannheim, Germany).

CSF Aβ_1–42_, tau, and ptau were measured by commercially available ELISAs (Innotest -β-amyloid_(1-42)_, Innotest hTAU-Ag 168 and Innotest Phosphotau_(181P)_; Innogenetics, Ghent, 169 Belgium). The performance of the assays was monitored with internal quality controls consisting of pools of surplus CSF specimens. In the study period, multiple internal quality controls with various concentrations have been used. The interassay coefficient of variation (CV) (mean ± standard deviation) was 11.3 ± 4.9% for Aβ, 9.3 ± 1.5% for tau, and 9.4 ± 2.5% for ptau [21].

### Angiotensin-converting enzyme protein assays

A commercially available sandwich enzyme-linked immunosorbent assay (R&D Systems, Abingdon, UK) was used according to the manufacturer’s guidelines to measure the ACE concentration in CSF and serum, as described previously [22]. Absorbance was read at 450 nm on a FLUOstar OPTIMA plate reader (BMG Labtech, Aylesbury, Buckinghamshire, UK). The samples were analyzed in duplicate, and ACE concentrations were interpolated from the standard curves of known concentrations of recombinant human ACE (929-ZN; R&D Systems). The ACE inhibitor captopril was purchased from Enzo Life Sciences (Exeter, UK). The interassay CV (mean ± standard deviation) was 8.7 ± 8.9% for CSF ACE and 13.0 ± 7.0% for serum ACE. The intra-assay CV was 7.9 ± 6.5% for CSF ACE and 7.6 ± 5.6% for serum ACE.

### Angiotensin-converting enzyme activity assays

Monoclonal anti-human ACE antibody (MAB929), recombinant human ACE (929-ZN) and the internally quenched fluorogenic peptide substrate (Mca-RPPGFSAFK(Dnp)-OH; ES005) were purchased from R&D Systems and were used to optimize an immunocapture-based fluorogenic assay for measurement of ACE activity in CSF and serum (see also Additional file 1) based on the method previously used by Miners and colleagues [23]. Fluorescence was measured by excitation at 320 nm and emission at 405 nm, in a FLUOstar OPTIMA plate reader. The mean fluorescence of the negative controls was subtracted from standard and sample readings, as was done for the fluorescence of the ACE-specific captopril-inhibited well. The ACE-specific inhibitor captopril inhibited ACE activity by more than 90%. Each sample was measured in duplicate and the mean fluorescence of ACE activity was calculated, based on the standard curve of recombinant ACE. The interassay CV (mean ± standard deviation) was 8.5 ± 8.4% for CSF ACE and 8.1 ± 8.7% for serum ACE. The intra-assay CV was 8.7 ± 5.9% for CSF ACE and 9.1 ± 8.5% for serum ACE.

### Data analyses

We used multiple imputation (10 datasets) to address missing values (see Table 1), and data were analyzed using SPSS version 20.0 (Chicago, IL, USA), by pooling the 10 imputed datasets [24,25].

**Table 1:** Baseline characteristics for the separate study groups after imputation

	**Control group (*n*** **= 40)**	**AD group (*n*** **= 78)**
Demographics		
Age (years)	63 (8)	68 (7)**
Female sex (%)	47	64*
Education (range 1 to 7)	6 (3 to 7)	5 (3 to 6)
*APOE*-ϵ4 carrier (%)	23	66*
MMSE (range 0 to 30)	28 (26 to 30)	22 (16 to 27)*
Vascular risk factors/disease		
Smoking (% current)	23	23
Body mass index (kg/m^2^)	25 (5)	24 (5)
Diabetes mellitus (%)	14	14
Hypercholesterolemia (%)	28	22*
Blood pressure measures		
Systolic (mmHg)	133 (14)	147 (22)**
Diastolic (mmHg)	83 (9)	86 (13)**
Hypertension (%)	30	55*
ACE measures		
CSF ACE protein level (ng/ml)	3.78 (1.30)	3.56 (1.16)**
CSF ACE activity (RFU)	155 (61)	148 (58)*
Serum ACE protein level (ng/ml)	4.22 (1.50)	3.72 (1.14)**
Serum ACE activity (RFU)	255 (152)	205 (116)**
CSF biomarkers		
Amyloid β_1–42_ (pg/ml)	887 (622 to 1,157)	475 (334 to 743)**
Total tau (pg/ml)	246 (122 to 371)	619 (344 to 1,102)**
Phosphorylated tau (pg/ml)	45 (29 to 74)	87 (54 to 137)**

Data presented as mean (standard deviation), percentage, or median (10th to 90th percentile). Percentage of missing values before imputation: education, 0.8%; *APOE* genotyping, serum ACE protein and activity levels, 1.7%; MMSE, 3.4%; smoking, 7.6%; diabetes mellitus and hypercholesterolemia, 14%; all other variables, 0%. ACE, angiotensin-converting enzyme; AD, Alzheimer’s disease; APOE, apolipoprotein E; CSF, cerebrospinal fluid; MMSE: Mini-Mental State Examination; RFU, relative fluorescence units.*P < 0.05 versus controls, **P < 0.01 versus controls.

First, patient characteristics were calculated for separate study groups (control group and AD group) and differences were evaluated by analysis of variance for continuous variables, or by Fisher’s exact test for categorical variables. Second, Pearson’s correlation coefficients between ACE protein and activity levels for each of CSF and serum in turn were examined. Pearson’s correlation coefficients between Aβ and tau or ptau were also analyzed. Third, we examined the linear regression between *APOE*-ϵ4 allele and Aβ, tau and ptau (Model 2). Fourth, linear regression analysis was used to investigate the associations of ACE measures (continuous) with log-transformed CSF biomarker levels of Aβ, tau and ptau (continuous), unadjusted (Model 1) and adjusted for age, sex and study groups (Model 2). Since inheritance of the AD risk-associated *APOE*-ϵ4 allele and vascular risk factors including blood pressure measures could influence the association between ACE measures and CSF biomarkers, we additionally adjusted for *APOE*-ϵ4 (carrier vs. noncarrier) and vascular risk factors (systolic blood pressure, diastolic blood pressure, body mass index, smoking, history of hypercholesterolemia or diabetes mellitus) (Model 3). Finally, analysis of covariance was used to estimate the adjusted mean log-transformed CSF Aβ, tau and ptau levels across tertiles of ACE measures within the separate study groups. For graphical purposes, the log-transformed values were back transformed.

## Results

In the total population of 118 subjects, the mean age was 66 (standard deviation, range: 8, 50 to 79) years and 52% were female. Table 1 shows that patients in the AD group were older, more often female, and more often *APOE*-ϵ4 carriers than patients in the control group. They also had less hyperlipidemia and higher blood pressure levels. CSF Aβ levels were lower in AD patients, and CSF tau and ptau levels were higher in AD patients. Further, all ACE measures were lower in patients with AD compared with patients in the control group (Table 1). The Pearson correlation coefficient between CSF ACE protein level and CSF ACE activity was 0.26 (*P* = 0.005), between serum ACE protein level and serum ACE activity was 0.28 (*P* = 0.002), between CSF and serum ACE protein levels was 0.54 (*P* < 0.001), and between CSF and serum ACE activity levels was 0.07 (*P* = 0.494). The correlation between tau and Aβ was -0.43 (*P* < 0.001) and between ptau and Aβ was -0.41 (*P* = 0.0004). Further, *APOE*-ϵ4 carriers had significantly lower Aβ (Β = -0.32), and significantly higher tau (Β = 0.50) and ptau levels (Β = 0.31); *P* < 0.001 for all.

### Cerebrospinal fluid ACE measures and cerebrospinal fluid biomarkers

**Figure 1:**
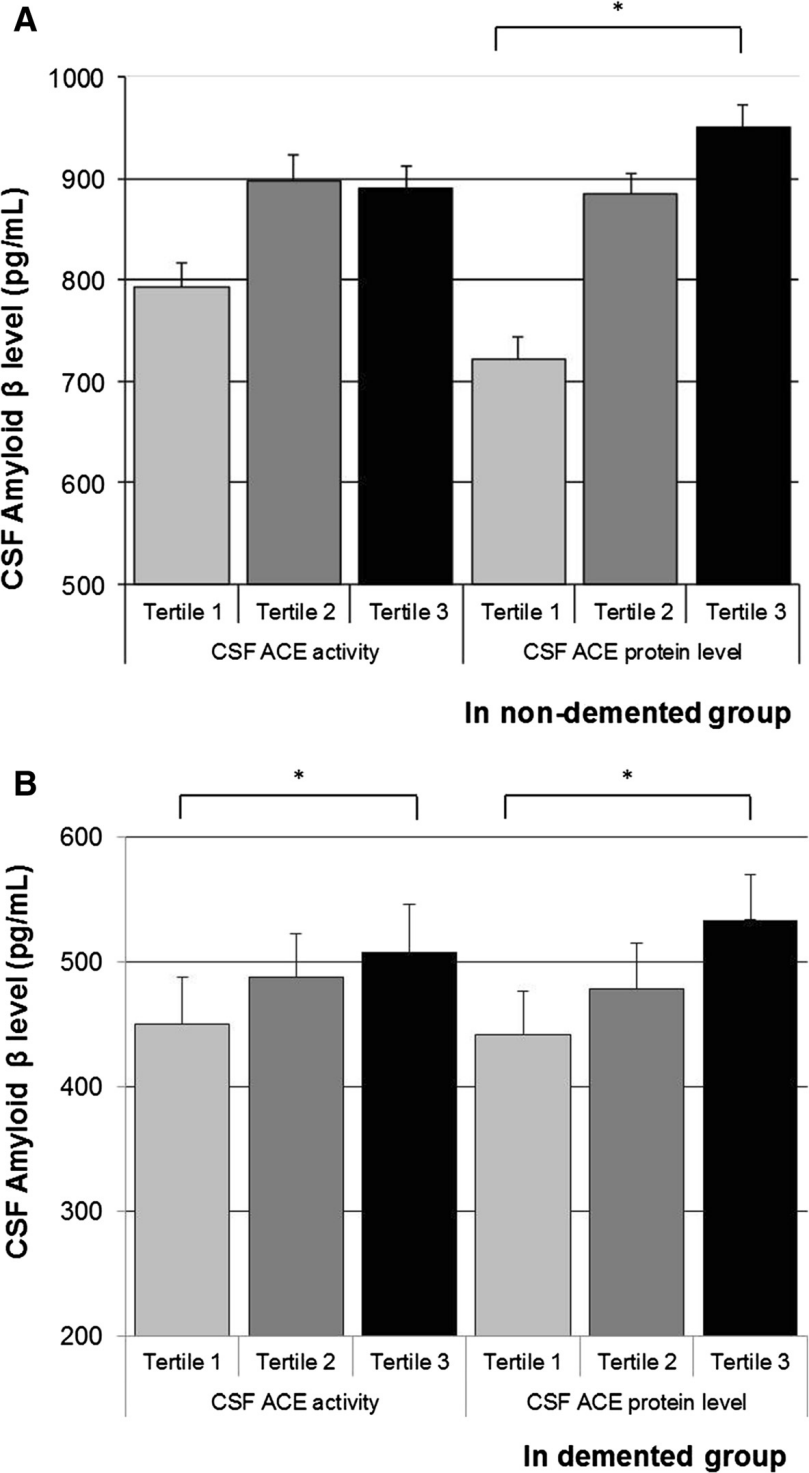
**Angiotensin-converting enzyme measures and cerebrospinal fluid amyloid-β**_**1–42**_**.** Mean (standard error) in cerebrospinal fluid (CSF) amyloid-β_1–42_ across tertiles of CSF angiotensin-converting enzyme (ACE) activity and CSF ACE protein level, in the control group **(A)** and the demented group **(B)**. Analyses were adjusted for age and sex. Tertiles for CSF ACE activity: <121.66; 121.66 to 177.56; >177.56 relative fluorescence units. Tertiles for CSF ACE protein level: <2.95; 2.95 to 3.92; >3.92 ng/ml. *P < 0.05.

**Figure 2:**
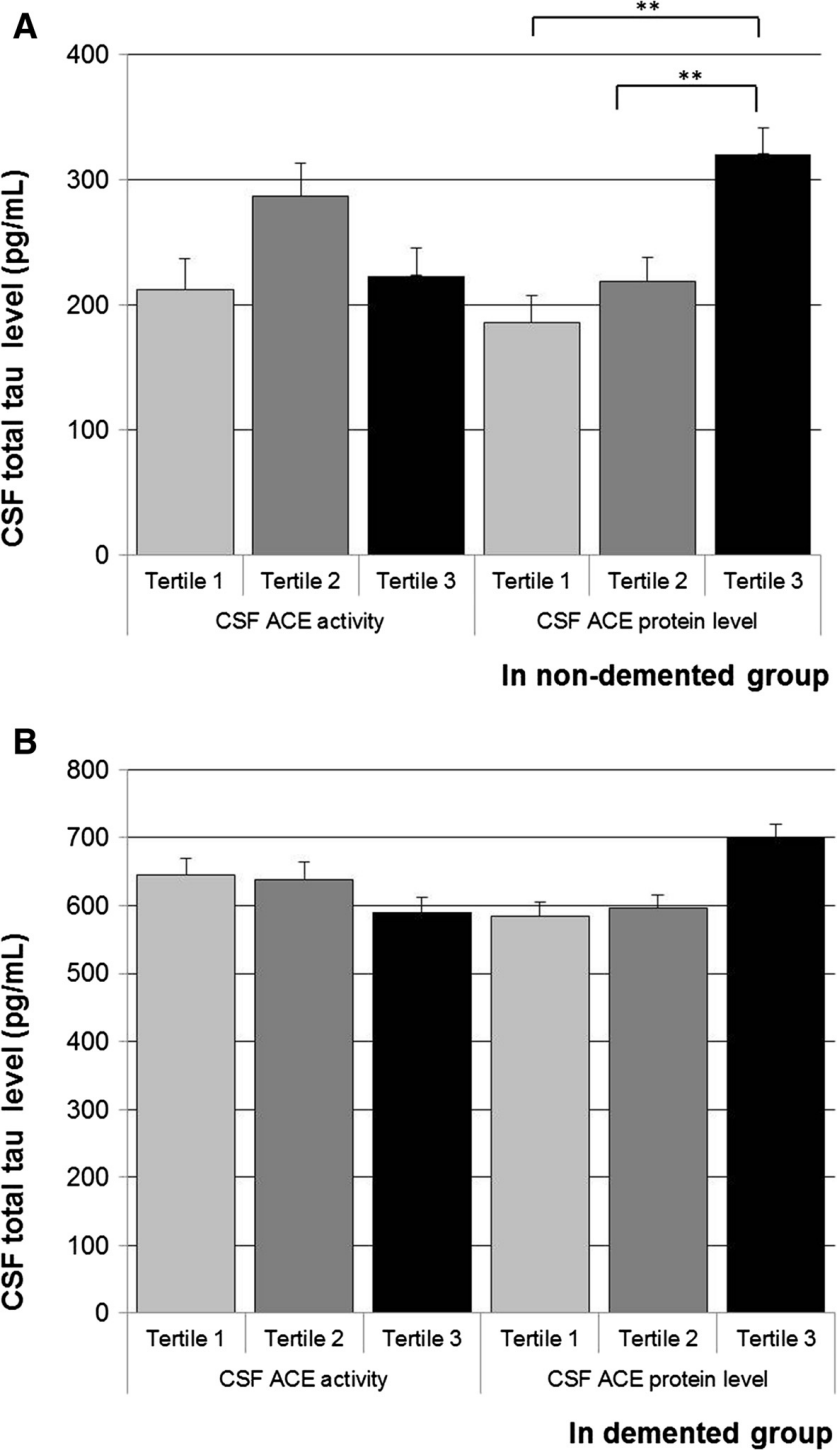
**Angiotensin-converting enzyme measures and cerebrospinal fluid total tau.** Mean (standard error) in total tau across tertiles of cerebrospinal fluid (CSF) angiotensin-converting enzyme (ACE) activity and CSF ACE protein level, in the control group **(A)** and demented group **(B)**. Analyses were adjusted for age and sex. Tertiles for CSF ACE activity: <121.66; 121.66 to 177.56; >177.56 relative fluorescence units. Tertiles for CSF ACE protein level: <2.95; 2.95 to 3.92; >3.92 ng/ml. **P < 0.01.

**Figure 3:**
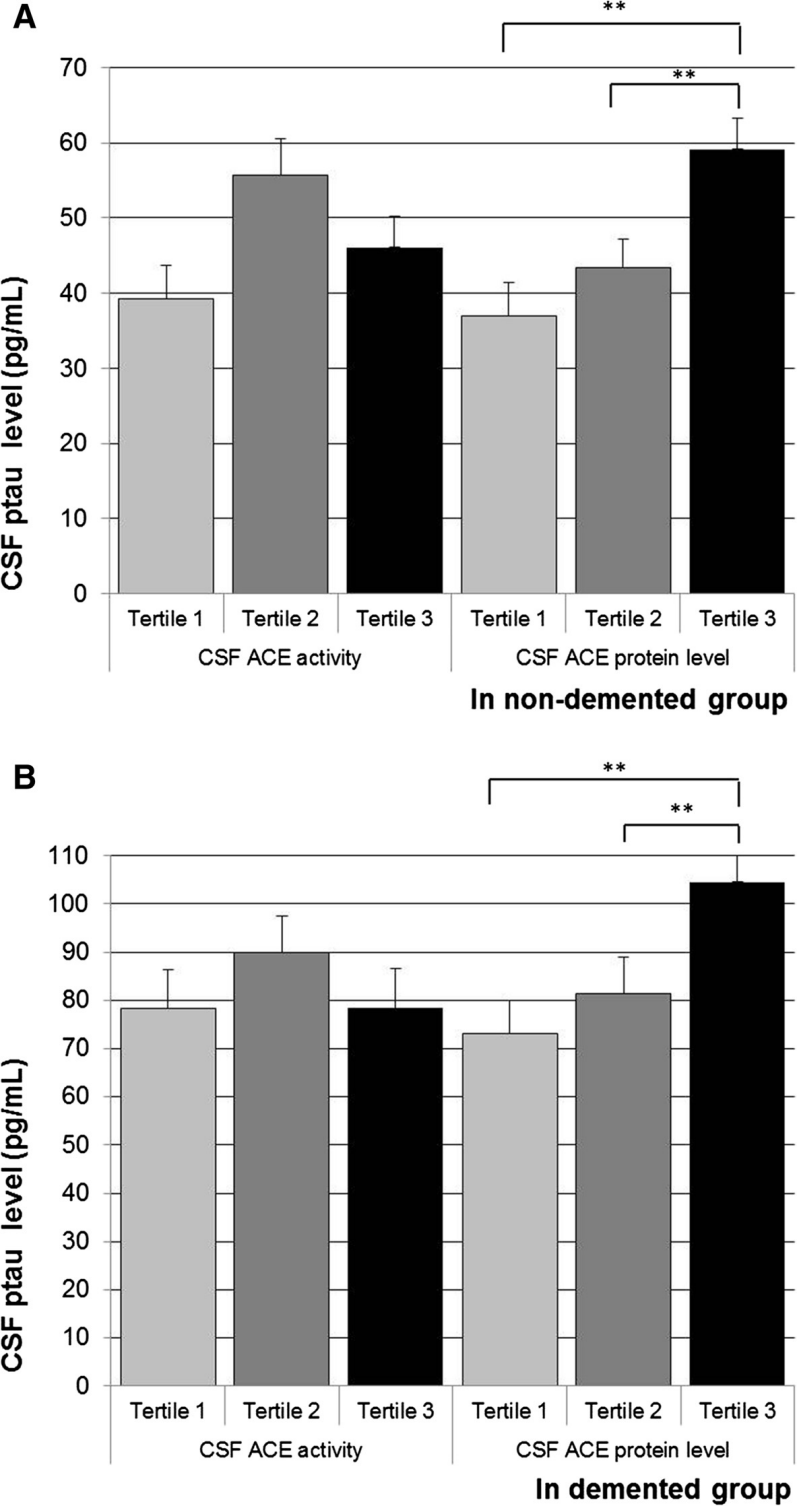
**Angiotensin-converting enzyme measures and cerebrospinal fluid phosphorylated tau.** Mean (standard error) in phosphorylated tau across tertiles of cerebrospinal fluid (CSF) angiotensin-converting enzyme (ACE) activity and CSF ACE protein level, in the control group **(A)** and the demented group **(B)**. Analyses were adjusted for age and sex. Tertiles for CSF ACE activity: <121.66; 121.66 to 177.56; >177.56 relative fluorescence units. Tertiles for CSF ACE protein level: <2.95; 2.95 to 3.92; >3.92 ng/ml. **P < 0.01.

In the total population, higher CSF ACE protein level was independently associated with higher CSF Aβ, tau and ptau levels (Table 2). When stratified by study group, the associations between higher CSF ACE protein levels and higher CSF Aβ, tau and ptau were present in the control group and the AD group, although significance was lost for CSF tau in the control group (Figures 1, 2 and 3).

**Table 2:** **Linear regression analyses between CSF and serum ACE measures and log-transformed CSF biomarkers in the total population (*n*** **= 118)**

	**Model**	**Ln Aβ42**	**Ln tau**	**Ln ptau**
CSF ACE protein level^a^	1	0.12 (0.05; 0.19)**	0.10 (-0.01; 0.22)	0.14 (0.05; 0.22)**
	2	0.09 (0.04; 0.15)**	0.15 (0.06; 0.25)**	0.17 (0.10; 0.25)**
	3	0.11 (0.05; 0.16)**	0.16 (0.05; 0.26)**	0.19 (0.10; 0.27)**
CSF ACE activity^b^	1	0.07 (-0.00; 0.14)	-0.06 (-0.18; 0.06)	0.02 (-0.07; 0.11)
	2	0.05 (-0.00; 0.11)	-0.03 (-0.13; 0.07)	0.04 (-0.04; 0.12)
	3	0.06 (0.00; 0.11)*	-0.04 (-0.14; 0.06)	0.03 (-0.05; 0.11)
Serum ACE protein level^c^	1	0.09 (0.02; 0.17)*	-0.09 (-0.21; 0.03)	-0.02 (-0.11; 0.07)
	2	0.06 (-0.00; 0.12)	-0.03 (-0.13; 0.07)	0.02 (-0.06; 0.10)
	3	0.04 (-0.02; 0.11)	-0.04 (-0.15; 0.06)	0.02 (-0.06; 0.11)
Serum ACE activity^d^	1	0.06 (-0.01; 0.14)	-0.10 (-0.22; 0.01)	-0.02 (-0.10; 0.07)
	2	0.03 (-0.03; 0.09)	-0.05 (-0.15; 0.05)	0.02 (-0.06; 0.10)
	3	0.02 (-0.04; 0.08)	-0.06 (-0.17; 0.05)	0.01 (-0.08; 0.10)

Data presented as Β (95% confidence interval). Model 1: unadjusted. Model 2: adjusted for age, sex and study groups. Model 3: additionally adjusted for *APOE*-ϵ4 genotype, systolic blood pressure, diastolic blood pressure, body mass index, smoking, history of hypercholesterolemia and diabetes mellitus. Aβ, amyloid-beta; ACE, angiotensin-converting enzyme; CSF, cerebrospinal fluid; Ln, log-transformed; ptau, tau phosphorylated at threonine 181; RFU, relative fluorescence units; tau, total tau. *P < 0.05, **P < 0.01. ^a^Per standard deviation increase (1.32 ng/ml). ^b^Per standard deviation increase (59 RFU). ^c^Per standard deviation increase (1.30 ng/ml). ^d^Per standard deviation increase (133 RFU).

Higher CSF ACE activity was also marginally associated with higher CSF Aβ levels, but not with CSF tau or ptau levels (Table 2). When stratified by study groups, the association was particularly present in the AD group (Figure 1).

### Serum ACE measures and cerebrospinal fluid biomarkers

In the total population, higher serum ACE protein level was associated with higher CSF Aβ (borderline significance), but not with CSF tau or ptau levels (Table 2). When stratified by study groups, the association was more evident in the control group and not in the AD group: mean differences (95% confidence interval) between the highest and lowest serum ACE protein tertiles were 193 (47; 340) and 44 (-62; 151), respectively (Model 2).

Serum ACE activity was not associated with higher CSF Aβ, tau or ptau levels (Table 2).

## Discussion

The main findings of this study are that lower CSF ACE protein levels, and to a lesser extent serum ACE protein and CSF ACE activity levels, were associated with lower CSF Aβ levels, indicating increased Aβ accumulation in the brain. Surprisingly, lower CSF ACE protein levels were associated with lower CSF tau and ptau levels, indicating less brain tau pathology. These associations were found in both control and AD patients and were independent of age, sex, *APOE* genotype, and vascular risk factors. Further, CSF and serum ACE protein and activity levels were lower in AD patients compared with controls.

To our knowledge, this is the first study that linked direct measures of ACE activity to the CSF AD biomarkers Aβ, tau, and ptau. Our results on the association of CSF ACE protein level and CSF Aβ are in line with studies that showed an association between the *ACE* gene and Aβ. Haplotypes of the *ACE* gene that were associated with lower ACE levels were related to lower CSF Aβ [13,14], and another study showed that the *I*-allele (indicating lower ACE levels) was associated with slightly more Aβ load in the brain at autopsy [26]. The Rotterdam study examined the *ACE* genotype in relation to brain atrophy (which is associated with increased brain Aβ plaques) [1], and found that female *I/I* genotype carriers had smaller hippocampal and amygdalar volumes [27]. Further, we showed in the SMART-MR study that lower serum ACE levels were associated with more progression of cortical brain atrophy [28]. Together, these data suggest that lower ACE could lead to more Aβ accumulation in the brain, reflected by lower CSF Aβ, and support previous reports that higher ACE levels, through degradation of Aβ, may contribute to less accumulation of Aβ into senile plaques in the brain and subsequently be beneficial for the occurrence of brain atrophy.

Another novel finding was our observation that lower CSF ACE protein level was associated with lower CSF tau and ptau. A previous study relating the *ACE* genotype to tau load in the brain found no association [26]. The mechanisms explaining the relation between lower ACE and lower CSF tau levels remain largely unknown, although a role for ANGII could be suggested. A recent study showed that central infusion of ANGII in normal rat brains induced tau phosphorylation in a dose-dependent manner via activating glycogen synthase kinase 3β. Furthermore, this was reversed by co-administration of the ANG II type 1 receptor antagonist, losartan, which is commonly prescribed for the treatment of hypertension [29]. Our findings suggest that higher ACE levels which lead to higher ANGII levels can induce tau pathology in the brain, and in turn result in higher tau levels in the CSF. Coincidentally, and perhaps ironically, while such elevations in ACE may exacerbate tau-related pathology, these same elevations may also have a slightly beneficial mitigating effect in terms of reducing Aβ pathology. Yet no association was seen between CSF tau and ptau with CSF ACE activity, which we might expect to be a more biologically meaningful measure of the likely production of ANGII from its precursor ANGI [17].

CSF ACE levels were more strongly associated with AD biomarkers than serum ACE levels. This indicates that CSF ACE, as might be expected, could reflect the activity of the brain RAS, thereby showing stronger associations with brain pathology. Further, ACE protein levels were more strongly associated with AD biomarkers than ACE activity levels. Whereas protein levels indicate the concentration of ACE in body fluid, activity levels particularly indicate the ANGI to ANGII conversion capacity of the enzyme [22]. One might thus have expected that ACE activity levels in particular would be more indicative of and relevant to Aβ degradation, and would therefore be more strongly associated with CSF Aβ. At present we cannot explain this apparent inconsistency; it could be partly related to the *ACE* genotype although the genetic contribution to ACE levels has only been shown to be relatively modest [12], and unfortunately we did not have information on the *ACE* genotype in our population so we could not explore this. Another reason might be differential levels of post-translational modification in the various physiological compartments from which the samples were derived. ACE activity, and its proposed conversion of Aβ, has been shown previously to be modulated by post-translational modification [30]. In both CSF and serum there may therefore be differential levels of glycosylation that can explain the lower than expected correlations of activity with some of our outcome measures. Miners and colleagues previously showed a trend of divergence between ACE levels and activity in postmortem CSF, but this was not significant and the patterns observed may have been an artifact of postmortem delay where additional changes to the ACE may have occurred post mortem [22]. Yet here remains the possibility that in the brain, from which the levels of Aβ, tau and ptau emanate, levels of ACE activity are influenced in a more disease-specific manner by advancing pathology than occurs in the more peripheral compartments we have tested. Indeed, Miners and colleagues have shown in postmortem brain tissue from AD patients that their ACE activity and not their ACE levels are more markedly different than in the control group [22]. Similarly, according to our data there is some support that disease-specific modifications of ACE may be involved since the apparent discrepant findings between ACE levels and activity with respect to CSF Aβ were less marked and seemed to correlate more in the AD group (Figure 1B).

Among the strengths of the current study is the use of CSF ACE measures and CSF biomarkers, which gives the opportunity to examine during life the association between ACE and AD pathology, without potential confounding from postmortem delays. Second, we had paired CSF and serum samples for all of the patients. Finally, all patients were assessed in a standardized way and diagnosed according to commonly used criteria [18].

One of the limitations of our study is the relatively small study population. Yet the interesting results recommend replication in a larger study sample. Another limitation is the cross-sectional design that prohibits conclusions on cause or effect. Similarly, we cannot ascertain whether lower CSF ACE protein and activity levels cause brain Aβ pathology. However, because we found that the associations were also present in the control group, it is reasonable to assume that the downregulation of CSF ACE is not pathology driven, and that lower ACE levels might precede the pathology or alternatively are a general phenomenon associated with neurodegeneration. This hypothesis remains to be further investigated in a larger, longitudinal study.

This study has important clinical implications. If ACE in the brain does prevent Aβ accumulation in living humans, our data support the suggestions that taking ACE inhibitors as antihypertensive drugs, particularly those that can cross the blood–brain barrier, might compromise Aβ degradation [31]. Fortunately, while uncertainty remains on this issue, angiotensin-receptor blockers, which solely target ANG II effects whilst not interfering directly with ACE, could provide an immediate and readily available potential alternative anti-hypertensive therapy for patients at risk for AD [31-33].

## Conclusion

We found in a memory clinic cohort that lower CSF ACE levels were associated with lower CSF Aβ levels, which suggests more accumulation of Aβ in the brain. This observation lends support to the hypothesis that ACE can degrade Aβ, thereby contributing to limiting its accumulation and possibly to the development or rate of progression of AD.

## Abbreviations

Aβ: amyloid-beta; ACE: angiotensin-converting enzyme; AD: Alzheimer’s disease; ANG: angiotensin; *APOE*: apolipoprotein E; CSF: cerebrospinal fluid; CV: coefficient of variation; ptau: tau phosphorylated at threonine 181; RAS: renin–angiotensin system; tau: total tau; VUMC: VU University Medical Center.

## Competing interests

PGK has undertaken some advisory work for Novartis, which was paid to the University of Bristol. The remaining authors declare that they have no competing interests.

## Authors’ contributions

HMJ, CET, MM, MIG, WMvdF and PS made substantial contributions to the conception and design, and were involved in drafting the manuscript or revising it critically for important intellectual content. ELA, CET, PGK, REJ, HMJ and MM made substantial contributions to the acquisition of data, or the analysis and interpretation of data. All authors gave final approval of the version to be published.

## Supplementary Material

Additional file 1 Is a description of the ACE activity assays. Click here for file
