# Supplementary material for: The association of angiotensin-converting enzyme with biomarkers for Alzheimer’s disease
Source: Alzheimers Res Ther. 2014 May 15;6(3):27. doi: 10.1186/alzrt257 (PMC4075229; doi:10.1186/alzrt257)
Supplement: Additional file 1 — Is a description of the ACE activity assays. [file alzrt257-S1.docx]

**Additional file 1: ACE activity assays**

Monoclonal anti-human ACE antibody (MAB929), recombinant human ACE (929-ZN) and the internally quenched fluorogenic peptide substrate (Mca-RPPGFSAFK(Dnp)-OH) (ES005) were purchased from R&D systems (Abingdon, UK). Captopril was purchased from Enzo Life Sciences (Exeter, UK). Wells of a NuncMaxiSorp 96-well polystyrene immunoplate (Fisher Scientific, Loughbourgh, UK) were coated with capture monoclonal anti-human ACE antibody (1 µg/ml) diluted in PBS (pH 7.4), the plate sealed and incubated for 18 hours at room temperature. Wells were washed five times in PBS (pH 7.4) containing 0.05% tween-20 (Sigma Aldrich, Dorset, UK) and non-specific binding of antibody blocked by incubating with 300 μl of PBS (pH 7.4) containing 1% bovine serum albumin (BSA) (Fisher Scientific) for 2 hours at room temperature. Serial dilutions of recombinant human ACE (2500 - 40 ng/ml) were prepared in PBS (pH 7.4) to create a standard curve of enzyme activity. For sample wells, 10 µl of CSF or 15 µl of serum was diluted in PBS (pH 7.4) (100 µl/well), prepared in triplicate. After washing the plate five times, diluted recombinant human ACE and diluted samples were briefly vortexed and added to required wells. Control wells were included on each plate and contained 100 µl PBS alone. The plate was sealed and incubated for 2 hours at room temperature with continuous shaking. After a further five washes, an optimal concentration of a specific inhibitor peptide, captopril (1mM) prepared in distilled water was added to required wells (50 µl/well) and distilled water was added to all uninhibited wells (50 µl/well). The plate was sealed and incubated for 10 minutes at room temperature prior to the addition of fluorogenic substrate (10 µM) prepared in 100 mM Tris-HCl pH 7.5 containing 50 mM NaCl and 10 µM ZnCl_2_ to all wells (50 µl/well). The plate was sealed and incubated in the dark for 2 hours with continual shaking at 37°C, followed by incubation at 4°C for 16 hours. Fluorescence was measured with excitation at 320 nm and emission at 405 nm, in a FLUOstar OPTIMA plate reader (BMG Labtech, UK) after 18 hours incubation.
